# Bacterial contamination on clinical surfaces and oxygen device accessories in the emergency unit of a tertiary health facility in Ghana

**DOI:** 10.1186/s12879-023-08894-6

**Published:** 2024-01-02

**Authors:** Evans Thompson, Akua Tutuwaa Badu, Emmanuella Abban, Evelyn Baawa Eyeson, Leslie Larry Afutu, Bless Amankwaah, Suzzana Dickson Buabeng, Abigail Agyen Frimpong, Alberta Serwah Anning, George Ghartey-Kwansah

**Affiliations:** 1 Department of Biomedical Sciences, College of Health and Allied Sciences, University of Cape Coast, Cape Coast, Ghana; 2 Cape Coast Teaching Hospital, Cape Coast, Ghana; 3 Cocoa Clinic, Kejebril-Takoradi, Apowa Road, Takoradi, Ghana

**Keywords:** Bacterial contamination, Oxygen device accessories, Emergency unit, Clinical surfaces, Tertiary health facility

## Abstract

**Background:**

Nosocomial infections have gradually become an emerging threat to the healthcare system over the past decades and have been attributed to poor decontamination of hospital articles and weak antibacterial stewardship policies. This study sought to investigate the effect of disinfection on the prevalence and resistance profile of bacterial contaminants on oxygen device accessories, and clinical surfaces at the emergency unit of a tertiary health facility in Ghana.

**Methods:**

The study employed a cross-sectional study design to evaluate the occurrence of bacteria on surfaces in a tertiary hospital. Luminal swabs of the oxygen device accessories and swabs from clinical surfaces used by healthcare providers were collected for isolation and identification of bacteria. The identified bacteria isolates were then tested for their susceptibility to antibacterial agents. Data from this study were analyzed using Excel (Microsoft Office Suite), and GraphPad Prism 8 software programs.

**Results:**

A quarter of the total 44 bacterial isolates obtained from both post-disinfected and pre-disinfected surfaces were Gram-positive, with the remaining isolates being Gram-negative. *Pseudomonas aeruginosa* was the most frequent bacteria species isolated (41%) followed by *Citrobacter sp.* (21%). *P. aeruginosa, S. aureus, and S. pneumoniae* were found to be highly resistant to Chloramphenicol (36%), and Sulfamethoxazole (100%); whereas Ciprofloxacin (91%) was the most effective antibacterial drug used.

**Conclusion:**

The almost equal prevalence of multidrug-resistant bacteria from both post-disinfected and pre-disinfected surfaces of inanimate objects, and oxygen device accessories connote an ineffective disinfection process which may influence resistance in bacterial contaminants. This requires the overhaul of disinfection protocol and training of hospital staff, and rational use of antibacterial agents at the hospital to mitigating the burden of nosocomial infections.

## Background

Nosocomial infections or healthcare-associated infections (HAIs) refer to diseases that occur in patients receiving medical care in hospitals, which were absent at the time of admission. These infections can occur during healthcare attainments or even after patients are discharged [1]. Microbes have an inherent ability to colonize any surface, and studies have shown that microbes can persist for weeks on stainless steel, and polymeric materials used to fabricate touch surfaces in hospitals. The longer a nosocomial pathogen persists on a surface, the longer it may be a source of transmission to susceptible patients or healthcare workers [2].

Oxygen therapy is vital in treating patients with critical breathing difficulty, usually due to an underlying disease condition in the patient such as chronic obstructive pulmonary disease, or when the ambient oxygen is lower than required for normal internal physiologic activities [3]. In such life-threatening situations where hypoxia needs to be avoided for any fatality from necrosis, supplementary administration of oxygen becomes not just one of many alternatives but a must [4, 5].

The nasopharyngeal area has a rich microbiota which is disseminated into the environment through respiration. During oxygen therapy, the propulsive mechanism of respiration drives air and portions of dislodged normal flora into the oxygen tubes, seeding them with the organisms [6]. Humidification provides ambient support for bacterial growth in the oxygen device accessories, and nose masks used in the clinical administration of artificial oxygen. Even though microbes can be destroyed by disinfection procedures, they can persist and be viable in the lumen of the tubes when exposed to ineffective decontaminants. Thus, reusable oxygen therapy gadgets can harbor these microbes and infect other patients [7].

The increase in HAIs can also be caused by improper decontamination methods used in most healthcare settings, especially in emergency units. Surfaces such as tables, bedsides, books, equipment, disposables, and reusables serve as reservoirs for microbes; and if not properly decontaminated, pose serious threats to patients' mortality and morbidity. According to Cornejo-Juarez et al., 2008, multi-drug resistance may arise from poorly treated HAIs and can lead to obnoxious outcomes if immediate interventions are not utilized [8]. Antibacterial resistance occurs when microbes advance in mechanisms that protect them from the effect of the antibacterial agent [9]. This may be due to irrational use of antibacterial agents, especially in patients with acute infections that require the use of broad-spectrum antibiotic [10].

The antibacterial stewardship practice in the hospital is a major element of combating antibacterial resistance. Clinical microbiologists can play key roles in the implementation of antibacterial stewardship programs through their contribution to antibacterial susceptibility reports with the ultimate aim to improve antibiotic use and optimize the treatment of infection in critically ill patients [11].

Although there is a paucity of data on microbial contamination of commonly touched surfaces and healthcare devices in Ghanaian secondary and tertiary healthcare facilities, it is worth arguing that these objects are often contaminated and colonized by antimicrobial-resistant bacteria regardless of disinfection and sterilization [12–14]. The presence of bacteria on medical and healthcare devices has been shown to cause HAIs and deaths in patients, including respiratory complications due to prolonged mechanical ventilation [15–17].

This study sought to investigate the effect of disinfection on the prevalence and resistance profile of bacterial contaminants on oxygen device accessories and clinical surfaces at the emergency unit of a tertiary health facility in Ghana. Findings from this study will serve as the basis for the health facilities in Ghana to review their disinfection protocols and policies. It will also provide physicians with information on the treatment of HAIs in patients admitted to the hospital facility, and contribute to scientific knowledge while making recommendations for further research on hospital-acquired infections in Ghanaian hospitals.

## Methods

### Sample collection

A total number of sixteen (16) swabs were collected from the emergency unit of the tertiary health facility; eight (8) from accessories of one oxygen device and the remaining eight (8) from clinical surfaces in the emergency unit. The sampled items included a humidifier, nose mask, low-flow nasal cannula, high-flow nasal cannula, patients’ locker, trolley, bedside, and a nurse’s table. A sample was collected pre-disinfection and post-disinfection using swab sticks moistened with normal saline. Swab sticks were placed back into the containing tube and transported on ice to the laboratory for analysis.

### Processing and identification of isolates

Sample processing and identification of the isolates were performed per the standard operating procedures (SOPs) of the laboratory. The samples were cultured on the routinely used microbiological media (Nutrient Agar) and incubated at 37 °C. If no growth was observed after 24 h, the plates were further incubated for a total of 48 h [18]. The isolates were identified based on colony morphology, Gram stain, and standard confirmatory biochemical tests. Gram-positive bacteria were identified by testing the hemolytic activity on blood agar and further identified using different biochemical tests such as catalase reaction, slide and tube coagulase tests, bile esculin, in addition to diverse differentiating antibiotic discs such as optochin and bacitracin. Identification of Gram-negative bacteria was done based on biochemical tests such as oxidase, triple sugar iron, motility indole ornithine, citrate, lysine iron arginine, and urease tests [18, 19].

### Antimicrobial susceptibility

Antibacterial susceptibility tests were performed using the Kirby-Bauer disk diffusion method, and interpreted according to the 33rd Edition of the Clinical Laboratory Standard Institute (CLSI) guidelines. A standardized suspension of bacteria (0.5 McFarland standard) was inoculated onto Mueller Hinton Agar (MHA) in a petri dish, after which filter paper disks impregnated with a standardized concentration of antibacterial agents (Chloramphenicol 30 µg, Sulfamethoxazole 23.75 µg, Meropenem 10 µg, Ciprofloxacin 5 µg) were placed on the surface and incubated for 24 h at 37 °C [20].

### Data analysis

The data from this study were stored and analyzed using Microsoft Excel and GraphPad Prism 8 respectively. The analysis was performed using the z-test with a 95% confidence interval. The data from the analysis were presented in graphs and tables to show the distribution of isolates, and antibacterial susceptibility profiles.

## Results

Findings from this study show that there is no significant difference in bacteria prevalence on surfaces of oxygen device accessories and clinical surfaces post-disinfection (Table 1 z-test, *P* > 0.05, CI: -2.250 to 2.250). Bacterial contaminants isolated from oxygen device accessories were diverse and included *Pseudomonas aeruginosa, Citrobacter sp., Staphylococcus aureus, Staphyolococcus epidermidis, Yersina sp., Corynebacterium sp., Moraxella catarrhalis, Neisseria meningitidis, Streptococcus pneumoniae, and Haemaphilus influenzae* while contaminating species isolated from the common surfaces included only *Pseudomonas aeruginosa, Citrobacter sp., and Listeria sp.*

**Table 1:** Distribution of bacteria isolates on clinical surfaces and oxygen-delivery devices

Bacteria species	Common Surfaces		Oxygen device accessories	
	Post-disinfected (%)	Pre-disinfected (%)	Post-disinfected (%)	Pre-disinfected (%)
*Pseudomonas aeruginosa*	6 (27)	6 (27)	3 (13)	3 (13)
*Citrobacter sp.*	3 (14)	4 (18)	2 (9)	0
*Listeria sp.*	2 (9)	1 (5)	0	0
*Staphylococcus aureus*	0	0	3 (13)	2 (9)
*Staphylococcus epidemidis*	0	0	1 (5)	0
*Yersinia sp.*	0	0	1 (5)	0
*Cornebacterium*	0	0	1 (5)	0
*Moxarella catarrhalis*	0	0	0	2 (9)
*Neisseria meningitidis*	0	0	0	1 (5)
*Streptococcus pneumoniae*	0	0	0	2 (9)
*Haemaphilus influenza*	0	0	0	1 (5)

Face masks, humidifiers, nurses’ tables, and lockers were the most contaminated surfaces before and after disinfection with *P. aeruginosa* as the most prevalent contaminant as opposed to the less frequent *Neisseria meningitides, Haemophilus influenza, Corynebacterium sp., Listeria sp.,* and *Yersinia sp*. (Table 1). Similarly, *Pseudomonas aeruginosa* (26%) was highly prevalent on both post-disinfected and pre-disinfected surfaces of the oxygen device accessories followed by *Staphylococcus aureus* (13%) which was observed to be widely distributed on the post-disinfected device accessories.

*Citrobacter sp., S. epidermidis, Yersinia sp., and Corynebacterium sp.* were isolated only from the post-disinfected surfaces. *Moraxella catarrhalis, N. meningitidis, S. pneumoniae,* and *H. influenzae* were isolated from the pre-disinfected surfaces only (Table 1). *P. aeruginosa* and *Staphylococcus aureus* were the most resistant to the various antibiotics tested. Also, the majority of the bacteria isolates were resistant to the antibiotic Sulfamethoxazole, an indication that the drug had little bacteriostatic effect on the isolates (Table 4).

### Prevalence of bacteria on oxygen device accessories

Bacteriological analysis showed that there were no substantial changes in the bacterial contamination of oxygen device accessories after disinfection, except for the nose mask which produced fewer bacterial isolates post-disinfection. Comparing the number of bacteria isolates obtained from samples before and after disinfection, it could be deduced that the disinfection practice did not drastically reduce bacterial contamination. Table 2 shows the frequencies of isolates identified on the various accessories of oxygen device accessories both before and after disinfection.

**Table 2:** Frequency of isolates obtained from both post-disinfected and pre-disinfected surfaces of oxygen device accessories

Post-disinfected oxygen delivery device				
Bacteria species	Mask	Humidifier	High flow canula	Low flow canula
*Staphylococcus aureus*	0	0	1	2
*Citrobacter sp.*	2	0	0	0
*Staphylococcus epidemidis*	1	0	0	0
*Yersinia sp.*	0	1	0	0
*Pseudomonas aeruginosa*	0	3	0	0
*Corynebacterium sp.*	0	0	1	0
Pre-disinfected oxygen delivery device				
*Staphylococcus aureus*	0	1	0	1
*Pseudomonas aeruginosa*	2	0	1	0
*Moraxella catarrhalis*	0	1	1	0
*Neisseria meningitidis*	0	1	0	0
*Streptococcus pneumoniae*	2	0	0	0
*Haemophilus influenzae*	0	0	0	1

### Antimicrobial susceptibility pattern of microbes

**Fig. 1:**
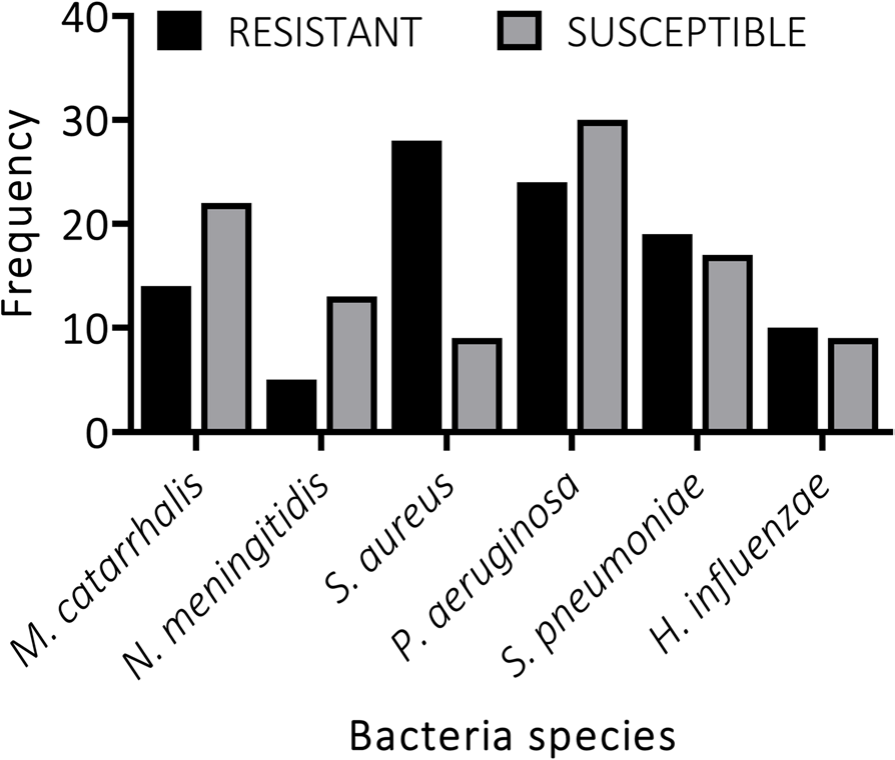
Percentage frequency of bacteria susceptibility and resistance patterns on pre-disinfected oxygen devices

**Fig. 2:**
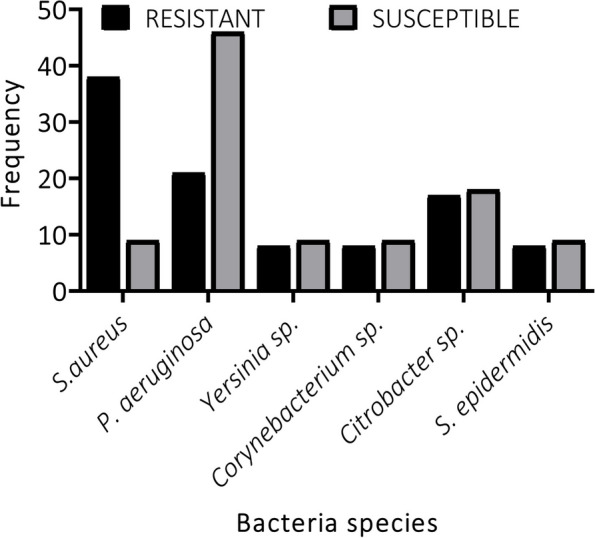
Percentage frequency of bacteria susceptibility and resistance patterns on post-disinfected oxygen devices

*Moraxella catarrhalis, N. meningitides*, and *P. aeruginosa* making 43% of isolates from pre-disinfected oxygen devices were susceptible to the antibiotics, whereas the remaining isolates showed either intermediate or complete resistance to the antibiotics (Fig. 1). This indicates a high resistance frequency among these bacteria. Interestingly, isolates from post-disinfected surfaces of oxygen devices showed similar patterns of resistance and susceptibility to the antibiotics; except for *S. aureus* and *P. aeruginosa* which were more resistant than susceptible, and more susceptible than resistant respectively (Fig. 2).

Data on susceptibility and resistance to specific antibiotics show similar patterns of resistance and sensitivity for both post-disinfected and pre-disinfected oxygen device accessories (Table 4). The bacteria isolates from the pre-disinfected oxygen devices were sensitive to Meropenem and Ciprofloxacin, but resistant to Sulfamethoxazole. Similar patterns were observed for isolates from post-disinfected oxygen devices; where bacteria isolates were susceptible to Meropenem, and Ciprofloxacin, except *S. aureus* isolated from the high and low flow cannula.

### Prevalence of bacteria on clinical surfaces

**Fig. 3:**
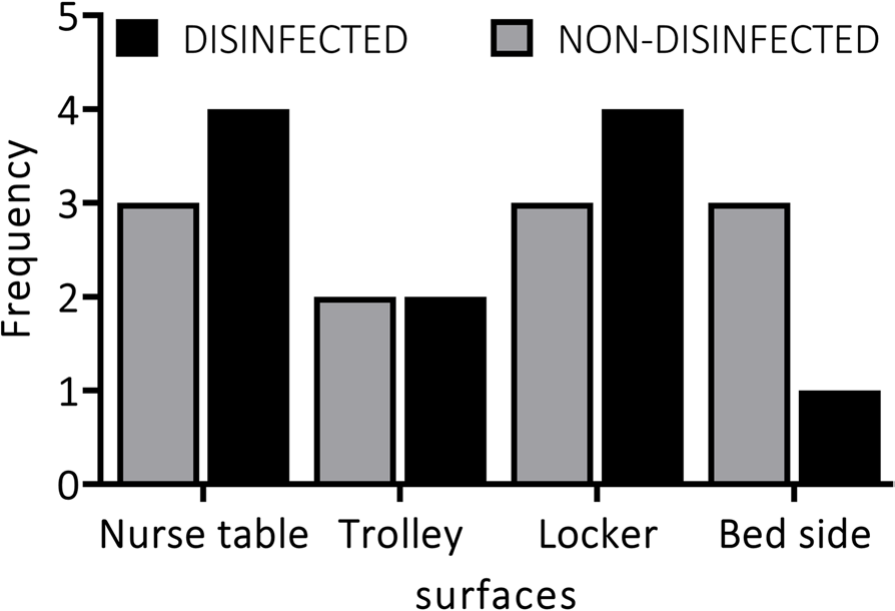
Frequency of isolates on both pre-disinfected and post-disinfected surfaces of clinical surfaces

The prevalence of bacteria on post-disinfected and pre-disinfected surfaces of commonly used items in the emergency unit was similar for all sampled surfaces, except for the bedside which produced fewer isolates post-disinfection as compared to several isolates before disinfection (Fig. 3). Samples taken from the nurses’ table and lockers post-disinfection produced more isolates than samples taken before disinfection. There was also an equal prevalence of bacteria before and after disinfection of the trolley used in the emergency unit (Table 3).

**Table 3:** Number of isolates obtained from both post-disinfected and pre-disinfected commonly used surfaces (fomites)

Post-disinfected commonly used surfaces			
Fomite	*Pseudomonas aeruginosa*	*Citrobacter sp.*	*Listeria sp.*
Nurses table	3	1	0
Trolley	2	0	0
Locker	1	1	2
Bedside	0	1	0
Pre-disinfected commonly used surfaces			
Nurses table	2	0	1
Trolley	0	2	0
Locker	1	2	0
Bedside	3	0	0

### Distribution of bacteria isolates on the common surfaces

**Fig. 4:**
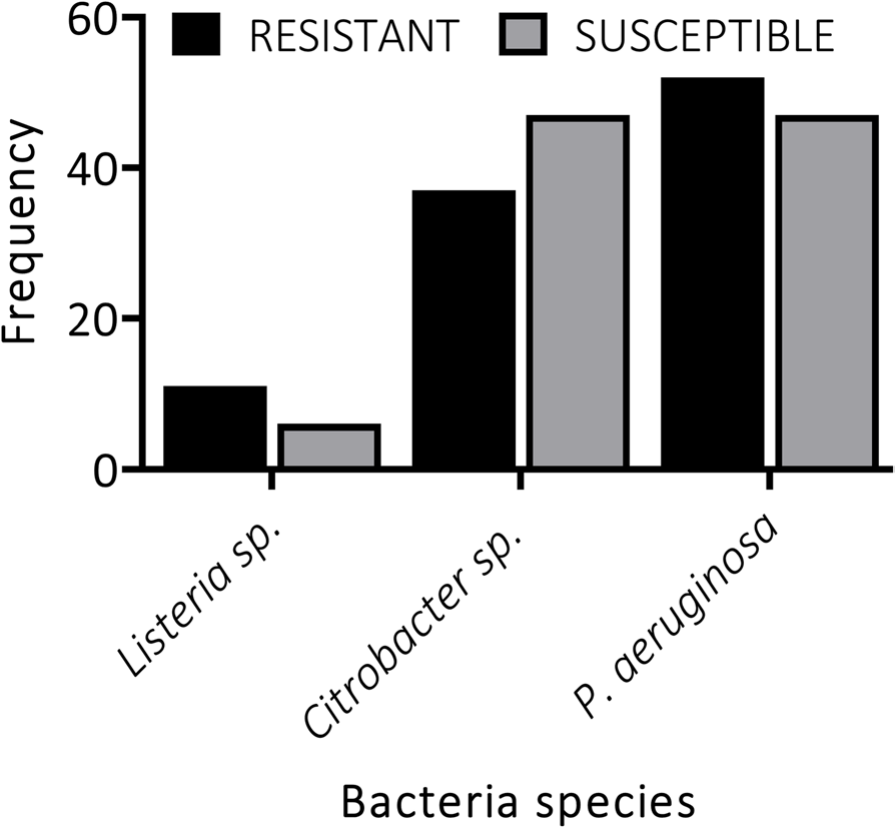
Percentage frequency of bacteria susceptibility and resistance patterns on non-disinfected common surfaces

As observed, *P. aeruginosa* was isolated at the same frequency in both post-disinfected (55%) and pre-disinfected (55%). *Citrobacter sp.* was identified more from pre-disinfected common surfaces (36%) than post-disinfected (27%), while twice the number of *Listeria sp.* were obtained from post-disinfected than pre-disinfected common surfaces (Table 1). In this case, *P. aeruginosa* and *Listeria sp.* obtained from the pre-disinfected surfaces were resistant to the antibiotics while *Citrobacter sp.* were susceptible to the antibiotics (Fig. 4).

### Antimicrobial susceptibility profile of bacteria

The antimicrobial profile of *Citrobacter sp., P. aeruginosa,* and *Listeria* sp. for both post-disinfected and pre-disinfected surfaces were similar, though with slight differences [Pre-disinfected: *Listeria sp.* (R = 5.3%, S = 2.6%), *Citrobacter sp*. (R = 18.4%, S = 23.7%), *P. aeruginosa* (R = 26.3%, S = 23.7%); Post-disinfected: *Listeria sp.* (R = 13.5%, S = 5.4%); *Citrobacter sp.* (R = 13.5%, S = 16.2%), and *P. aeruginosa* (R = 24.3%, S = 27.0%)]. All the bacteria isolates obtained from both post-disinfected and pre-disinfected clinical surfaces were susceptible to Ciprofloxacin, and resistant to Sulfamethoxazole. The susceptibility patterns of Meropenem and Chloramphenicol however were inconsistent – isolates from similar sources showed similar susceptibility and resistant patterns (Table 4). The isolates from post-disinfected clinical surfaces were resistant to Sulfamethoxazole, Meropenem, and Chloramphenicol, in order of decreasing resistance.

**Table 4:** Resistance and susceptibility patterns of isolates from both post-disinfected and pre-disinfected commonly used surfaces

Antimicrobial Resistance profile for isolates from commonly used surfaces				
Antibiotics	post-disinfected resistant	pre-disinfected resistant	post-disinfected susceptible	pre-disinfected susceptible
Meropenem	6	5	5	6
Sulfamethoxazole	11	10	0	0
Ciprofloxacin	0	0	11	11
Chloramphenicol	6	2	2	5

## Discussion

Findings from this study show that disinfection did not significantly reduce bacterial contaminants prevalent on the surfaces of oxygen delivery device accessories and clinical surfaces, and may influence the development of resistance in these bacterial contaminants. *P. aeruginosa* and *S. aureus* isolates showed higher resistance to the various antibiotics, whereas Sulfamethoxazole had little to no effect on a majority the isolates. At least one of the bacteria species isolated from oxygen device accessories and clinical surfaces were resistant to Ciprofloxacin and Meropenem.

Contamination of inanimate clinical surfaces may occur due to the shedding of bacteria from an infected patient or may be due to contamination from the hands of healthcare workers after direct contact with an infected patient [21], thus, the prevalence of bacteria on clinical surfaces even after disinfection. This could also be due to poor air quality since bacteria can be deposited by aerosols, dust, and other floating particles. The observed bacterial contamination due to antimicrobial and disinfectant resistant bacteria in this study poses the threat prolonged hospital stay and re-admissions to the emergency unit [15, 17]. Patients needing ventilators at the emergency unit, including the elderly and the critically ill, have a higher exposure to ventilation associated complications and mortality due to antibacterial resistant bacteria [6, 22].

Ineffective disinfection protocols or techniques in addition to intrinsic bacterial characteristics like biofilm formation could result in prolonged survival [23], accounting for the prevalence of bacteria on post-disinfected surfaces. It is important to keep healthcare settings clean to prevent the risk of exposing healthcare workers and patients, especially in emergency units to bacterial contaminants and reduce the risk of HAIs.

More than one bacteria species were isolated from both the post-disinfected and pre-disinfected surfaces, however, there was no significant difference in bacteria prevalence (*p*-value > 0.5). It was expected that the post-disinfected surfaces would harbor much lower contaminants, but according to Dvorak no single disinfection is effective against all microbes but should be effective to kill many bacteria species except those with spores [23, 24]. The relatively high prevalence of bacteria on post-disinfected surfaces suggests that either an ineffective disinfectant was used or the disinfection protocol was ineffective. 

The ineffective disinfection could be due to non-lethal concentrations of decontaminating agents or reduced contact time of bactericidal disinfectants imposing selective pressure on both commensal and pathogenic bacteria which gives rise to resistant species through vertical and horizontal transfer of resistant genes [25–27], resulting in phenotypic adaptation towards sub-lethal disinfectant concentrations. It could also be due to capsular membranes in the case of Gram-negative bacteria or the formation of biofilms which acts as a barrier preventing the uptake of disinfectants [21]. *Pseudomonas aeruginosa, Staphylococcus aureus, and Citrobacter sp*. have been reported by multiple studies to be resistant to disinfectants and antimicrobials [13, 28–30], and finding from this study agree with these reports because *Pseudomonas aeruginosa, Staphylococcus aureus, and Citrobacter sp*. were highly prevalent on fomites and oxygen device accessories and were less likely to be affected by disinfection compared to *Moraxella catarrhalis, Neisseria meningitidis, Haemophilus influenzae*. Thus, HAIs resulting from contamination would likely be an infection of *Pseudomonas aeruginosa, Staphylococcus aureus, or Citrobacter sp*.

The emergence of disinfectant resistance can be largely attributed to the abuse and misuse of disinfectants concomitant to a lack of understanding of the principles behind biosecurity [31, 32]. Thus, the efficacy of disinfection relies on different factors, including training and management of personnel on disinfection protocols [21].

Results of the bacteriological analysis show contamination of the oxygen device accessories by diverse bacteria species, whereas little diversity was observed for the clinical inanimate surfaces (Table 1)*.* The findings is corroborated by Suleyman and colleagues who reported *P. aeruginosa* as the most prevalent pathogen with the capacity to survive on dry surfaces of inanimate objects for months [33]. A different study also reported a higher prevalence (about 80%) of MRSA on bedside surfaces of intensive care units a few hours after disinfection with hypochlorite [34]. The frequent contamination of post-disinfected surfaces other than inherent resistant characteristics of the bacteria, can be due to a high touch frequency from patients and health attendants [35], as shown for the locker and nurses’ table. The diversity in bacteria isolates on the oxygen device accessories could be attributed to the shedding of nose and throat commensals by patients. The commensals isolated in this study, *Citrobacter sp., M. catarrhalis, H. influenza*, *S. aureus, N. meningitides*, and *P. aeruginosa,* though naturally inhabit the nose and throat, can cause serious life-threatening opportunistic diseases such as pneumonia, meningitis, septicemia, and urinary tract infection (UTI) [36, 37] in patients admitted to the emergency unit. These same bacteria species isolated in study have the potential to survive on dry surfaces for 2–24 h, *S. aureus* however can survive up to 8–21 days on the same surface [21]. Isolation of formerly mentioned species from pre-disinfected oxygen device accessories warrant critical evaluation of disinfection protocols of the health facility due to their pathogenic potential. Their potential to persist in oxygen device accessories after disinfection exposes patients with underlying conditions to the risk of developing respiratory complications.

Bacteria isolates from pre-disinfected surfaces showed higher resistance to antibiotics compared to isolates from post-disinfected surfaces. *Pseudomonas aeruginosa* isolates*,* despite the abundance and persistence, were largely more susceptible to the antibiotics with some degree of resistance towards Chloramphenicol, Sulfamethoxazole, and Meropenem. However, some studies reported *P. aeruginosa* to be more resistant to antibiotics instead [38, 39]. This difference in susceptibility can be attributed to local exposure to resistance factors including horizontal gene transfer. *Citrobacter sp., S. aureus*, *S. pneumoni*a, and* M. catarrhalis* were susceptible to Ciprofloxacin and Meropenem but resistant to Sulfamethoxazole and Chloramphenicol at varying frequencies.

the observed low resistance of the isolated species towards Ciprofloxacin could be due to the absence of resistant mechanisms against the agent. It is a broad-spectrum fluoroquinolone antibiotic which inhibits type II topoisomerase IV to inhibit mitosis in bacteria through DNA destruction, and these susceptible species may lack the necessary mutations or efflux pumps needed for resistance [31, 32, 40].

The high incidence of HCAIs and their associated mortalities is directly tied to the hospital environment (including inanimate objects), serving as a reservoir in the transmission of multi-drug resistant (MDR) bacteria [41, 42]. Findings from this study corroborate this fact and make an association between bacteria prevalence and disinfection. Healthcare providers may contribute to the spread of MDR bacteria in the emergency unit by carrying them to and from inanimate objects and infected patients.

## Conclusion

The near-equal prevalence of MDR-resistant bacteria from post-disinfected and pre-disinfected surfaces of inanimate objects, and oxygen delivery device accessories connotes an ineffective disinfection process which may have influenced the development of resistance in bacterial contaminants. Resistant bacteria contaminants can cause HAIs or exacerbate the conditions of patients admitted to the emergency unit. This warrants a review of disinfection protocol and training for health workers and janitors of major hospitals, and emphasis of rational use of antimicrobial agents since isolated bacteria were highly resistant to Sulfamethoxazole and susceptible to Ciprofloxacin. Effective infection transmission control requires a complete review of disinfection protocols, including the implementation of effective disinfection methods, and biosafety protocols with proper training of hospital staff. Future studies should scale up the study to investigate the prevalence of MDR bacteria in the entire hospital to determine how this relates to disinfection.

## Data Availability

The datasets generated and/or analyzed during the current study will be made available by the corresponding author on reasonable request, in accordance with the relevant data sharing policies of Springer Nature (BMC Infectious Diseases).

## Abbreviations

HAIs: Healthcare-associated Infections
MHA: Mueller Hinton Agar
CLSI: Clinical and Laboratory Standard Institute
BA: Blood Agar
TSI: Triple Salt Iron
WHO: World Health Organization
MDR: Multi-drug resistant
SOPs: Standard operating procedure
DNA: Deoxyribonucleic acid
MRSA: Methicillin-resistant Staphylococcus aureus
UTI: Urinary tract infection
